# Comparative transcriptome analysis reveals candidate genes for the biosynthesis of natural insecticide in *Tanacetum cinerariifolium*

**DOI:** 10.1186/s12864-016-3409-4

**Published:** 2017-01-09

**Authors:** Sana Khan, Swati Upadhyay, Feroz Khan, Sudeep Tandon, Rakesh Kumar Shukla, Sumit Ghosh, Vikrant Gupta, Suchitra Banerjee, Laiq ur Rahman

**Affiliations:** 1 Plant Biotechnology Division, Central Institute of Medicinal and Aromatic Plants (CSIR-CIMAP), Kukrail Picnic Spot Road, P.O. CIMAP, Lucknow, 226015 India; 2 Metabolic and Structural Biology Department, Central Institute of Medicinal and Aromatic Plants (CSIR-CIMAP), Kukrail Picnic Spot Road, P.O. CIMAP, Lucknow, 226015 India; 3 Process Chemistry and Chemical Engineering Department, Central Institute of Medicinal and Aromatic Plants (CSIR-CIMAP), Kukrail Picnic Spot Road, P.O. CIMAP, Lucknow, 226015 India

**Keywords:** *De novo* assembly, MEP pathway, MVA pathway, Oxylipin pathway, pyrethrins, *Tanacetum cinerariifolium*, Transcriptome, Unigenes

## Abstract

**Background:**

Pyrethrins are monoterpenoids and consist of either a chrysanthemic acid or pyrethric acid with a rethrolone moiety. Natural pyrethrins are safe and eco-friendly while possessing strong insecticidal properties. Despite such advantages of commercial value coming with the eco-friendly tag, most enzymes/genes involved in the pyrethrin biosynthesis pathway remain unidentified and uncharacterized. Since the flowers of *Tanacetum cinerariifolium* are rich in major pyrethrins, next generation transcriptome sequencing was undertaken to compare the flowers and the leaves of the plant *de novo* to identify differentially expressed transcripts and ascertain which among them might be involved in and responsible for the differential accumulation of pyrethrins in *T. cinerariifolium* flowers.

**Results:**

In this first tissue specific transcriptome analysis of the non-model plant *T. cinerariifolium*, a total of 23,200,000 and 28,500,110 high quality Illumina next generation sequence reads, with a length of 101 bp, were generated for the flower and leaf tissue respectively. After functional enrichment analysis and GO based annotation using public protein databases such as UniRef, PFAM, SMART, KEGG and NR, 4443 and 8901 unigenes were identified in the flower and leaf tissue respectively. These could be assigned to 13344 KEGG pathways and the pyrethrin biosynthesis contextualized. The 2-C-methyl-D-erythritol 4-phosphate (MEP) pathway was involved in the biosynthesis of acid moiety of pyrethrin and this pathway predominated in the flowers as compared to the leaves. However, enzymes related to oxylipin biosynthesis were found predominantly in the leaf tissue, which suggested that major steps of pyrethrin biosynthesis occurred in the flowers.

**Conclusions:**

Transcriptome comparison between the flower and leaf tissue of *T. cinerariifolium* provided an elaborate list of tissue specific transcripts that was useful in elucidating the differences in the expression of the biosynthetic pathways leading to differential presence of pyrethrin in the flowers. The information generated on genes, pathways and markers related to pyrethrin biosynthesis in this study will be helpful in enhancing the production of these useful compounds for value added breeding programs. Related proteome comparison to overlay our transcriptome comparison can generate more relevant information to better understand flower specific accumulation of secondary metabolites in general and pyrethrin accumulation in particular.

**Electronic supplementary material:**

The online version of this article (doi:10.1186/s12864-016-3409-4) contains supplementary material, which is available to authorized users.

## Background

Every year, 20–40% of the world’s crop production is lost to pests, weeds and diseases [1] and about $6000 is spent annually to combat these insects. Commonly used inorganic pesticides in agriculture have raised environmental and health concerns as these tend to persist in the environment for a long duration and thus continue to pose severe health hazards. The synthetic chemicals are non-biodegradable and highly toxic in nature and therefore 98% and 95% of the insecticides and herbicides respectively, are supposed to contribute to soil, water, and air pollution [2–4]. As a result biological species are continuously exposed to these hazardous surroundings. To address such problems, efforts have been made to look for alternative approaches for more eco-friendly means of controlling pests and insects including use of naturally occurring pyrethrins. Natural pyrethrins, offer many benefits over these chemicals like low toxicity, rapidity of action, active against a broad spectrum of insects, low costs, insect repellent, and no insect immunity. In addition, pyrethrins easily disintegrate in the air and sunlight, and are thus, considered as environmental friendly biodegradable compounds.

The *T. cinerariifolium* (previous species name: *Chrysanthemum cinerariaefolium*) aka pyrethrum, a perennial herb belonging to the family Asteraceae is a remarkable plant. The plant is well known for its economic importance as a source of an important group of secondary metabolites known as pyrethrins, which is a potent insecticide [5]. Pyrethrins are esters of either pyrethric acid or chrysanthemic acid with alcohol moiety termed as rethrolone [6]. Pyrethrins are a set of six structurally similar compounds including pyrethrin I, cinerin I, jasmolin I (pyrethrin Type I) and pyrethrin II, cinerin II, jasmolin II (pyrethrin Type II) [7]. Although pyrethrins occur throughout the aerial parts of the plant, the maximum accumulation of pyrethrin is concentrated in the flower heads, which is many folds higher than in leaves [8, 9]. According to USDA, pyrethrins and its synergists are considered as one of the safest and eco-friendly class of insecticides, which can be exploited at agro-industrial field levels [6]. Natural pyrethrins are directly extracted from the plant source with potential application for insect vector control. Pyrethrins are approved for such usage and also certified for use in organic gardening in the US [10].

As a prelude to enhancing the production of pyrethrins through targeted breeding programs, better understanding of the expression network of the genes and pathways associated with pyrethrin biosynthesis is required. Since the flowers and leaves differ substantially in the pyrethrin content, a facile insight could be obtained through comparing the transcriptome of the two organs of the plant. The next generation sequencing (NGS) approach of RNA seq using the Illumina platform has been widely adopted for transcriptome studies. However, to date, few full genome sequences are available in the Asteraceae family. This is attributed to heterozygosity, high chromosome number and the ploidy level of the genus Asteraceae where genome studies become more complicated [11]. Yet, a number of expressed sequence tags (ESTs) of various asteraceous species have been reported like gerbera hybrid, chicory [12, 13]. These would serve as a good source of gene comparison when taken together with the information available from model-plant genome sequences.

Although, there is considerable knowledge on the chemical structures and biochemistry of pyrethrins, the underlying molecular/biochemical mechanisms and the basis of variation in the bioactive constituents are still largely unknown. The percent content of pyrethrins in *T. cinerariifolium* flowers is governed by factors such as the plant genotype, ecological conditions and flower maturity [14, 15]. Earlier, the developmental gene/enzyme network in pyrethrin synthesis has been explored at the transcriptional level. In the present study the RNASeq-mediated transcriptome comparison of the *T. cinerariifolium* flowers and leaves was done, to obtain an insight into the genes involved in the biosynthesis of pyrethrins, not least because these genes are also involved in other secondary metabolite biosynthesis pathways. Based up on differential gene expression, the predicted candidate genes were found to be involved in pyrethrin/terpenes biosynthesis pathways. The unigenes and enzymes identified will lead to advancement in engineering of pyrethrin production in related species.

## Results

### Transcriptome sequencing, sequence quality control and *de novo* assembly

**Fig. 1:**
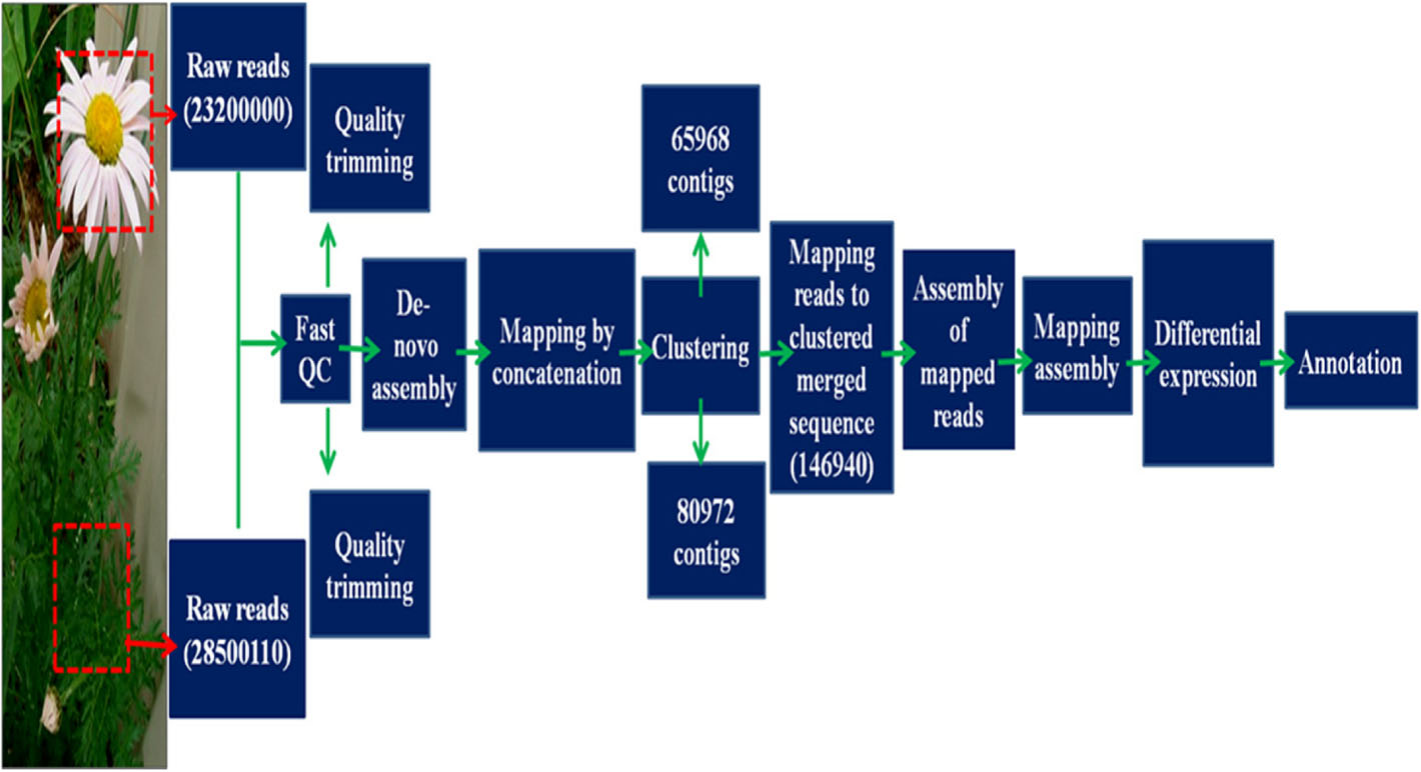
Strategy followed during the comparative analysis of Leaf versus flower Illumina transcriptome sequencing of *Tanacetum cinerariifolium*, data analysis, and functional annotation

Illumina 101 bp Paired-End sequencing run representing the cDNA library from leaf and flower tissue produced 23,200,000 reads for flower (PYT_F) and 28,500,110 reads for leaf (PYT_L) respectively. Total reads encompassed nearly 2.5 Gb of sequencing data in FASTAq format. Sequence data was filtered to remove low quality reads and reads containing adapter sequences. After quality control a total of 11,617,581 high quality sequencing reads for flower and 14,290,110 for leaf tissues remained, which were assembled into 65,968 and 80,972 unique sequences (contigs) for flower and leaf tissues, respectively. The lengths of these unique sequences were sufficient to enable functional annotations with high accuracy. The reads obtained were assembled by using Trinity software [16] (Fig. 1).

Several *de novo* assembly output parameters *de novo* were analyzed including total number of contigs, contigs with smallest length, N50 length, N80 length, longest contig length and smallest contig length as a function of k-mer. For above mentioned data set, N50 length was 575 and 1293, N80 length was 303 and 615, largest and smallest lengths were 8718 and 7717 and 201 and 201 for flower (PYT_F) and leaf (PYT_L) respectively. Total contig bases found for flower and leaf comprised 31.57e6 and 69.24e6 bp respectively.

### Gene ontology and functional annotation

A total of 65,968 and 80,972 unique sequences from the flower and leaf tissue were assigned with gene ontology (GO) terms based on sequence similarity to proteins in TAIR database. The *T. cinerariifolium* transcripts were assigned for GO terms to describe functions of genes and associated gene products into three, major categories namely; biological process, molecular function, and cellular component, and their sub-categories using plant specific GO that broadly provides an overview of the ontology content of the genes related to the pyrethrin biosynthetic pathway. The molecular function, biological process, and cellular component categories included 26190, 23862 and 22328 unigenes, respectively which were assigned into 34, 44 and 34 GO terms, respectively (Additional file 1).

**Fig. 2:**
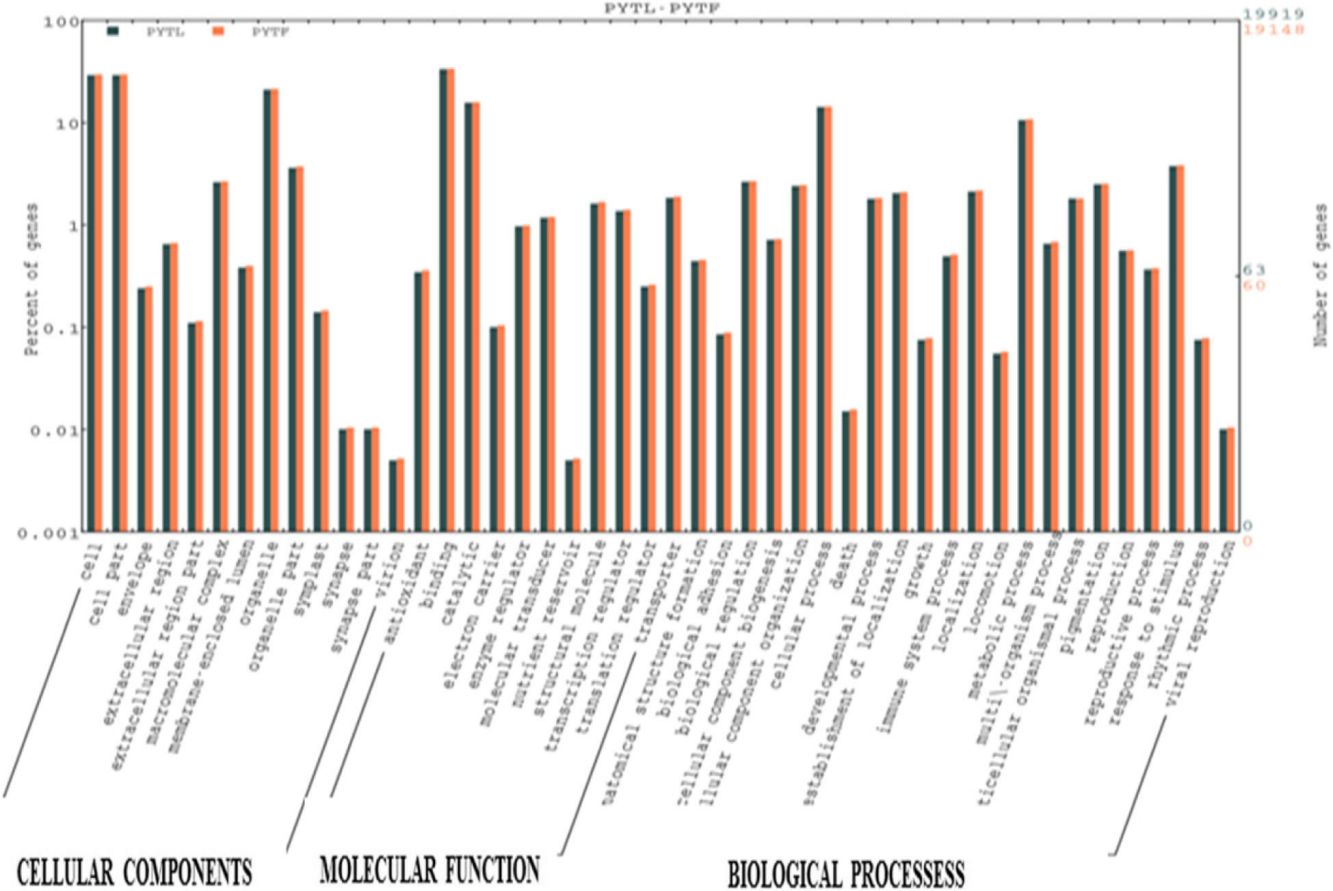
Histogram presentation of *T. cinerariifolium* unigenes among Gene Ontology functional classes. The results are categorized in three main categories: “Cellular component”, “Molecular Function” and “Biological process”. The left y-axis indicates the number of genes in particular category, and the left y-axis indicates the percentage of a specific category of genes in that main category

**Fig. 3:**
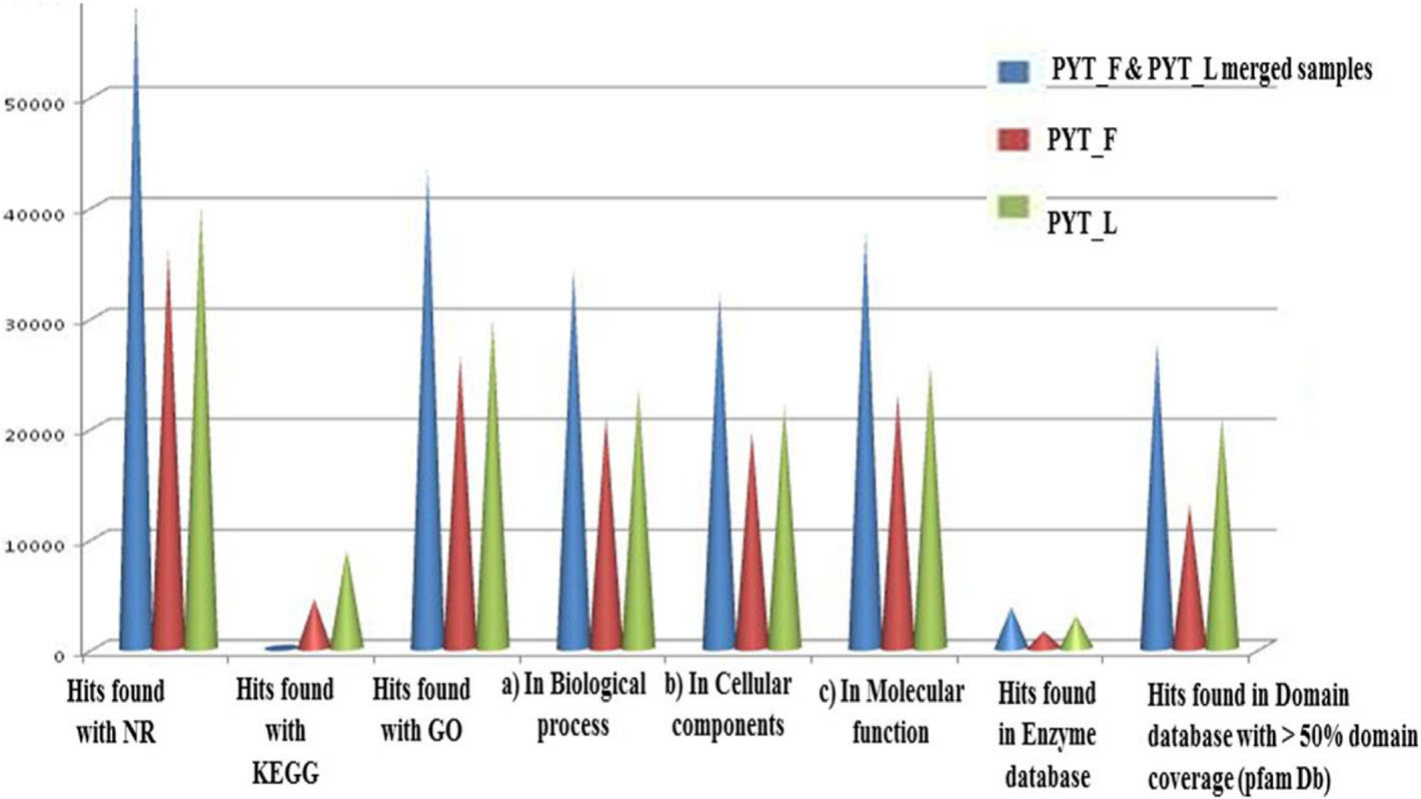
Annotation statistics (Top-HIT distribution of transcripts contigs generated by optimized parameters in the known databases) of leaf, flower and merged samples

**Fig. 4:**
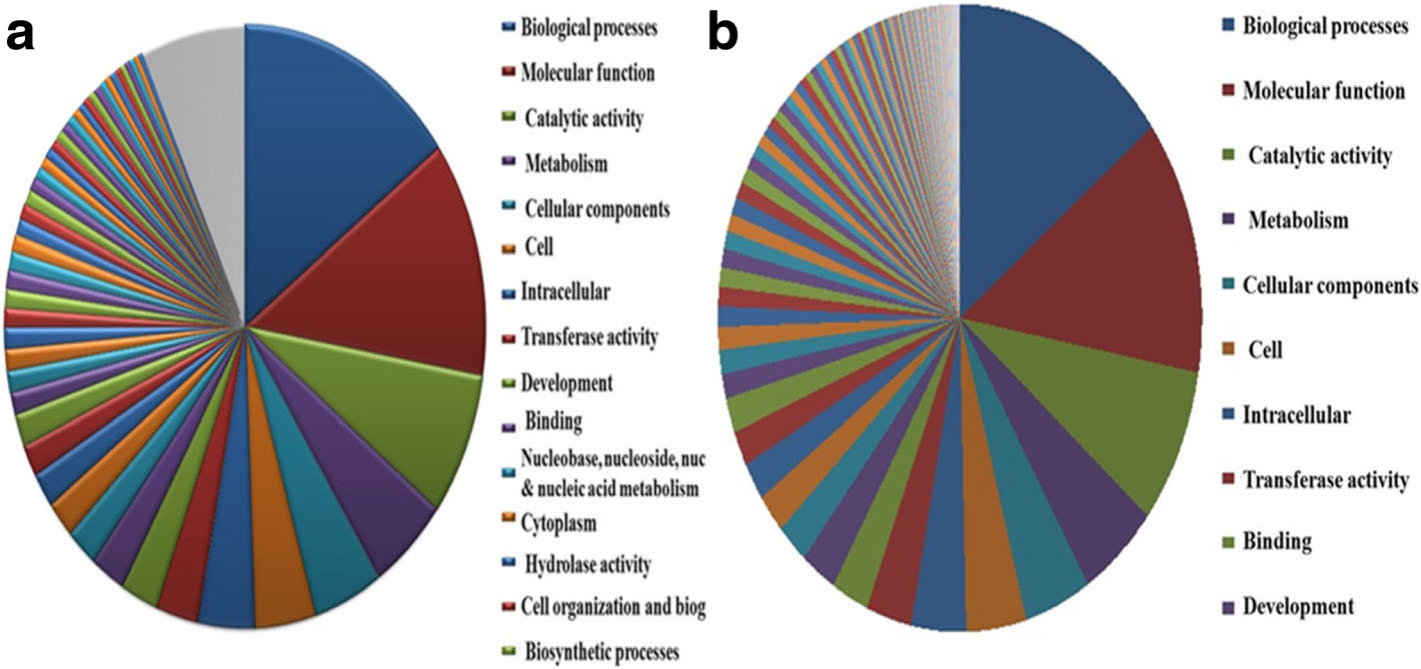
Pie chart showing proportions of transcripts classified based on GO in leaf and flower samples

In biological process group, 719, 283, 210 and 178 transcripts were assigned to metabolism, biosynthesis, nucleic acid metabolism and transport categories respectively. Similarly, 415, 381, 346 and 191 transcripts were assigned to cellular component cellular protein intracellular and cytoplasmic components categories respectively. In molecular function category, a total of 1409, 150, 363 and 271 transcripts were assigned to molecular activity, catalytic activity, transferase activity and hydrolase activity respectively (Additional files 2, 3, and 4; Fig. 2). These GO annotations provide a substantial information on potential functions of the transcripts identified in the *T. cinerariifolium* tissues. For the annotation and validation of the assembled unigenes, all the assembled unigenes were searched against the NCBI non-reduntant (Nr) and Swissprot protein databases using BLASTX program with an E-value threshold of 1^E-5^ (Fig. 3). A total of 9,304 unigenes were assigned to different GO category in leaf and flower tissues of *T. cinerariifolium.* Out of these 4558 unigenes belongs to flower and 4746 unigenes are found in leaf tissue separately (Fig. 4a, b).

### Comparison of proteome of *T. cinerariifolium* with the proteome of other plant species

**Fig. 5:**
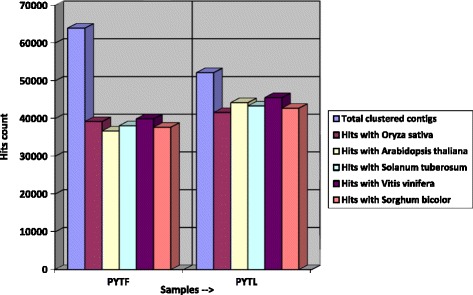
Bar chart showing proteome wide comparison of PYT_F and PYT_L with other plants proteome

To further evaluate the quality of the sequenced data, the comparison of the differential proteome data of *T. cinerariifolium* leaf and flower tissue with the published proteome data of other plants *viz; Arabidopsis thaliana*, *Sorghum bicolor*, *Vitis vinifera*, *Oryza sativa* and *Solanum tuberosum* was performed. Total 63,960 clustered transcripts (contigs) from flower were used for proteome comparison studies. Out of these contigs of flower, 39,214, 36,712, 38,084, 39,946 and 37,704 showed a match with *O. sativa*, *A. thaliana, S. tuberosum*, *V. vinifera* and *S. bicolor* respectively. Similarly, in leaf tissue 41629, 44246, 43379, 45554 and 42765 contigs showed match with *O. sativa, A. thaliana, S. tuberosum, V. vinifera* and *S. bicolor* respectively. Leaf tissue transcripts showed comparatively higher match with other plants. In both the tissues, maximum match was found with *Vitis vinifera* protein (Fig. 5).

### HPLC analysis

**Fig. 6:**
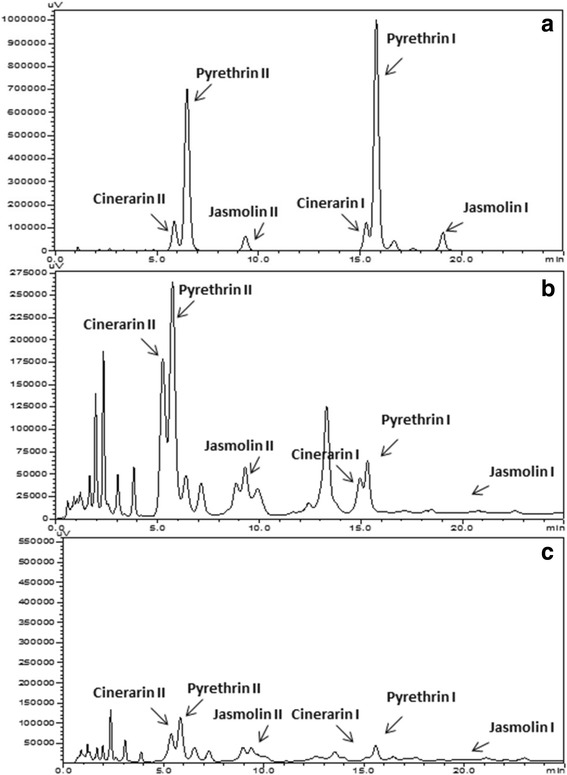
HPLC analysis showing six different pyrethrin peaks corresponding different pyrethrin compounds. Cinerin II, Pyrethrin II, Jasmoline II and Cinerin I, Pyrethrin I, Jasmoline I in (**a**) standard sample and (**b**) corresponding peaks in PYT_F and (**c**) showing chromatogram with less pyrethrin content in PYT_L respectively

High performance liquid chromatography was used to confirm the *de novo* biosynthesis of pyrethrin in flower verses leaf tissue of *T. cinerariifolium.* UV-visible absorption spectrum of flower and leaf extracts as well as of standard pyrethrin was recorded at 225 nm. The chromatograms of the standard pyrethrin and *T. cinerariifolium* (PYT_L and PYT_F) extracts recorded peaks corresponding to pyrethrin (Fig. 6). All pyrethrin esters were separated well in the sequential order as Cinerarin II, Pyrethrin II, Jasmolin II and subsequently followed by Cinerarin I, Pyrethrin I, Jasmolin I respectively.

### Metabolic pathway analysis through KEGG database

**Fig. 7:**
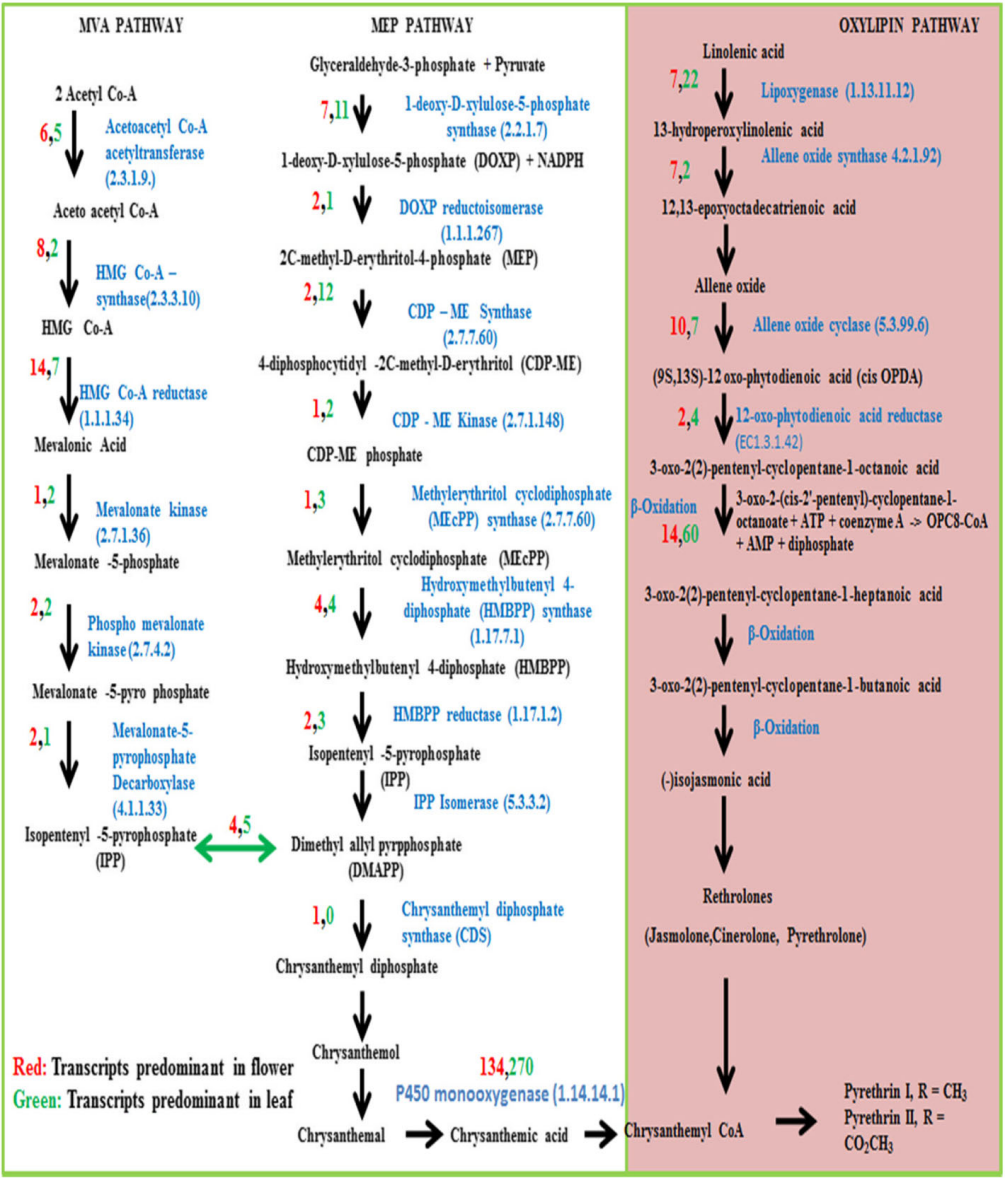
Proposed terpenoid biosynthetic pathway. HMG Co-A synthase (Hydroxymethylglutaryl-CoA synthase); HMG Co-A reductase (Hydroxymethylglutaryl-CoA reductase); DOXP reductoisomerase (1-deoxy-D-xylulose-5-phosphate reductoisomerase); CDP –ME synthase (2-C-methyl-D-erythritol 4-phosphate cytidylyltransferase); CDP-ME kinase (4-diphosphocytidyl-2-C-methyl-D-erythritol kinase); HMBPP reductase (4-hydroxy-3-methylbut-2-enyl diphosphate reductase), cppase (chrysanthemoyl pyrophosphate synthase). The numerical number in red represent predominant enzymes in flower and green represents enzymes predominant in leaf

In order to identify the biological pathways present in *T. cinerariifolium,* the assembled unigenes were annotated with corresponding enzyme commission (EC) numbers through BLASTX (NCBI, USA) analysis, against the (Kyoto Encyclopedia of Genes and Genomes) KEGG database [17]. Pathway analysis helped us to understand the presence of biological function and interaction of genes. After assembly, 4,443 unigenes in flower and 8,901 unigenes in leaf were found to have match with KEGG database and assigned to approximately 13344 KEGG pathways (Additional file 5). These data provide a valuable resource in finding out unigenes involved in secondary metabolic pathways specially terpenoid biosynthetic pathway for pyrethrin biosynthesis (Fig. 7).

### Identification and quantification of up/down regulated transcripts involved in pyrethrin biosynthetic pathways

The pyrethrin belongs to monoterpenoid backbone and constitutes two moieties; acid and alcohol moiety. The formation of acid (chrysanthemic/pyrethric) moiety utilizes both methylerythritol 4-phosphate (MEP) as well as mevalonate (MVA) pathways, however, the formation of rethrolones utilize oxylipin pathway. In studied transcriptome data, most of the unigenes related to pyrethrin biosynthesis pathway were successfully identified with their respective gene ontology.

The identified 291 enzymes (unigenes) in flower and 484 enzymes (unigenes) in leaves involved in pyrethrin biosynthesis pathway. Out of 565 unigenes, involved in MEP pathway; 211 and 354 upregulated transcripts in the flowers and leaves respectively were assigned for the formation of acid moiety of pyrethrin. Also 43 and 106 upregulated unigenes in flower and leaf respectively encoded enzymes involved in the oxylipin pathway, which forms the rethrolone moiety of the pyrethrin compound.

### Analysis of metabolic pathway genes which might be involved in pyrethrin biosynthesis

Biosynthesis of pyrethrin constitutes three pathways involving MVA, Oxylipin pathway and MEP pathway (Fig. 7). Total number of enzymes involved in MVA was more in flower (37 transcripts) as compared to leaf (24 transcripts). There are two steps working in the MEP pathway i.e. Step 1; the number of enzymes in leaf (36 transcripts) was higher than in flower (19 transcripts). Step 2 of MEP pathway the number of enzymes in flower (58 transcripts) was more than in leaf (48 transcripts). The enzymes involved in the oxylipin pathway of leaf were found to be expressed more than two folds than in flower. This result indicates that the potential candidate genes for pyrethrin synthesis in MEP and MVA pathways were more expressed in flower than in leaf. Although, the genes/enzymes involved in the synthesis of rethrolones were higher in leaf than in flower i.e. 106 and 43 respectively. In addition to this, 270 transcripts related to CyP450 were found to be expressed in leaves and 134 transcripts related to CyP450 with oxidoreductase activity were found to be expressed in flowers. The exact biosynthetic pathway involved in pyrethrin biosynthetic pathway *in planta* is not yet well known.

## Discussion

### *De novo* assembly and functional annotation

Despite a potentially major commercial value of the pyrethrins due to their utility as non-hazardous, eco-friendly pesticides and other pharmacological applications, the omics data on Asteraceae family are very limited. Some data is available in for Chrysanthemum species such as *C. morifolium* [18]. NCBI GenBank database contains 7,180 ESTs from Chrysanthemums. Here, the transcriptome comparison between the flowers and leaves for deeper understanding of pyrethrin synthesis and content enhancement adds to *de novo.*

The *de novo* transcriptome annotation studies on some important medicinal plants such as the Chinese fir [19], maize, safflower [20, 21], ramie [22], Emerald notothen [23] etc. In our study, total 23.2 million reads for PYTF (flower) & 28.5 million reads for PYTL (leaf) samples were used to assemble the flower and leaf transcriptome of *T. cinerariifolium*. The data obtained provides nearly 100% of high quality bases for both the flower and leaf tissues which reflected the high quality sequence run. The *de novo* assembly was generated using Trinity software [16]. The *de novo* assembly of *T. cinerariifolium* transcriptome was optimized after assessing the effect of various k-mer lengths. During the study, for assembly process, only those reads were considered that produced high frequency k-mer. In general, the longer the k-mer obtained, higher the proportionality to the accuracy of highly expressed transcripts in the genome. *de novo* Longer k-mers are advantageous to distinguish repeats from real overlaps while shorter k-mers preferred for assembly of low expression genes [24]. Further adapter sequences and low quality bases were trimmed. These results suggested that the transcriptome sequencing data of *T. cinerariifolium* were effectively assembled. The N50 and N80 values were higher which further suggests a better assembly.

Unigenes were assessed for a role in the KEGG database and were used to assign the functional GO annotation including cellular components, molecular functions and biological component group. This facilitated assigning the relevant genes to the secondary metabolite biosynthesis pathways. Mapping these unigenes we found the involvement of many (Fig. 7) in the biosynthesis of the pyrethrins either via universally present Oxylipin or MEP pathway.

### Identification of potential candidate genes involved in pyrethrin biosynthesis

**Fig. 8:**
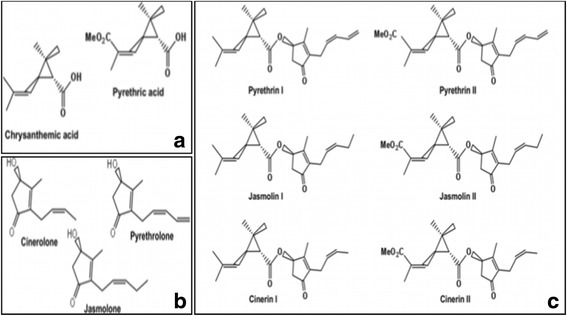
Major pyrethrins (I and II) reported from naturally occurring pyrethrin compound. **a** represents derivatives of acid moieties i.e. chrysanthemic acid and pyrethric acid. **b** represents derivatives of alcohol moieties i.e., cinerolone, pyrethrolone and jasmolone. **c** represents Pyrethrin I, Cinerin I, Jasmolin I and Pyrethrin II, Cinerin II, Jasmolin II

Pyrethrins are naturally occurred insecticides produced by certain species of chrysanthemum plants. Pyrethrins are accumulated in flowers but they are also synthesized in plant leaves [7, 25]. Pyrethrins are esters containing a combination of either chrysanthemic acid or pyrethric acid moiety with rethrolones as alcohol moiety (pyrethrolone, cinerolone, or jasmolone) (Fig. 8). The acid moieties are monoterpenes having a cyclopropane ring and are biosynthesized via 1-deoxy-D-xylulose 5-phosphate (DXP), which is formed by the condensation of pyruvic acid and glyceraldehydes-3-phosphate in the presence of 1-deoxy-D-xylulose S-phosphate synthase (DXS) enzyme. The cyclopropane ring formation is catalysed by chrysanthemyl diphosphate synthase (cppase) to give chrysanthemyl diphosphate using two molecules of dimethyl allyl pyrophosphate (DMAPP). The rethrolone moieties of pyrethrins are biosynthesized from linolenic acid via oxylipin pathway [7]. The most prominent types of pyrethrin are pyrethrin I and II. These are classified as terpenoids which are derived from cytosolic mevalonate (MVA) and plastidial methylerythritol 4-phosphate (MEP) pathway. Pyrethrins are more concentrated in the flower heads [8, 9]. Evidences support the involvement of both the biseriate and capitate glandular trichomes in the synthesis and storage of the pyrethrins [6, 9]. However, authentic evidences are still lacking in support of the synthesis and storage.

Most of the already known enzymes involved in the MVA pathway for monoterpene pyrethrin biosynthesis were found to be specifically expressed in the flower in comparison to leaf. Already reported literature suggested that the initial reactions of the pyrethrin biosynthetic pathway occured in the leaves, while the later modifications and its localization occur in the flower tissue and therefore the concentration of the pyrethrin is much more in the flowers. The identification of the transcripts responsible for *de novo* biosynthesis of pyrethrins from our current annotations was corroborated from the chromatograms suggesting that pyrethrins are majorly present in the flower as compared to leaf tissue of *T. cinerariifolium* from HPLC (Fig. 6).

There are two moieties involved in the process of biosynthesis of pyrethrin. The acid moieties are irregular monoterpenoid unit constitute a cyclopropane ring formed by the condensation of the two DMAPP molecules, the reaction is catalysed by chrysanthemyl diphosphate (CDS); a key enzyme, responsible for the conversion of chrysanthemic acid via intermediate precursor formation like chrysanthemol, chrysanthemal etc. Both the pathways i.e. MEP and MVA are responsible for the formation of DMAPP, in transcriptome analysis; it has been found that the number of candidate genes is higher in MEP pathway, operating in the plastid. On this basis it can be concluded that, DMAPP is predominately formed via MEP pathway and thus further utilized for the formation of acid moiety for the formation of pyrethrin.

Synthesis of chrysanthemic acid starts from glyceraldehyde -3-phosphate. The conversion of G-3-P to DOX and to MEP and further to 2-C-methyl-D-erythritol 4-phosphate cytidylyltransferase (CDP-MES) [E.C. 2.7.7.60] by 4-diphosphocytidyl- 2-C-methyl- D-erythritol kinase (CDP-MEK) [E.C. 2.7.1.148] (10 transcripts upregulated in leaf in our study) corresponds to the biosynthetic pathway for pyrethrins. Further conversion involves enzymes; 2-C-methyl-D-erythritol 2,4-cyclodiphosphate synthase, 4-hydroxy-3-methylbut-2-enyl-diphosphate synthase, 4-hydroxy-3-methylbut-2-enyl diphosphate reductase, isopentenyl-diphosphate delta-isomerase (MECPS [E.C.4.6.1.12]; HDS [E.C.1.17.7.1]; HDR [E.C.1.17.1.2]; IPP [E.C.5.3.3.2]; (3,4,3,5 upregulated transcripts in leaves respectively and 1,4,2,4 in flower respectively) that are specifically upregulated in leaves. According to the proposed pathway (shown in Fig. 7), some specific CYP450s and UDP-glycosyltransferases (UGTs) may catalyze the conversion of chrysanthemol and rethrolone to various Pyrethrins [26]. To date, no genes related to CYP450 that is involved in Pyrethrin biosynthesis have been identified from *T. cinerariifolium.* From transcriptome analysis the identified 270 cytochrome P450 [E.C.1.14.13.70] with monooxygenase activity is highly expressed specifically in leaves (270 transcripts) as compared to flower (134 transcripts), and hence, it can be hypothesized that acid moiety of pyrethrins in *T. cinerariifolium* is derived from MEP pathway. The cppase (chrysanthemyl diphosphate synthase); which is a key enzyme responsible for the *de novo* biosynthesis of pyrethrins is predominant in the flower tissue with a single transcript; however, in leaf tissue the enzyme was totally absent or no transcript was found (Fig. 7).

The alcohol moiety, rethrolone is chemically similar to plant hormone jasmonic acid. The biosynthesis of rethrolones starts with hydroxylation of linolineic acid catalysed by lipooxygenase (LOX, EC 1.13.11.12) (with 7 transcripts in flower, 22 transcripts upregulated in leaves) to give 13-hydroperoxylinolenic acid. The formation of 13-hydroperoxylinolenic acid, working as a substrate for the formation of allene oxide synthase (AOS, EC 4.2.1.92), which further catalyses the synthesis of unstable intermediate epoxyoctadecatrienoic acid and converted to allene oxide [27, 28]. The subsequent enzymes involved in the pyrethrin pathway like allene oxide cyclase (AOC, EC 5.3.99.6), responsible for the conversion of allene oxide to cis-OPDA, which is further exported to peroxisome where it is reduced to 3-oxo-2(2′-pentenyl)-cyclopentane-1octanoic acid via OPDA reductase. However, in the present transcriptomic data, the number of transcripts in flower (7 and 10 transcripts) was found higher than in leaves (2 and 7 transcripts) for AOS and AOC respectively. The further conversions involves various important enzymes (higher number of transcripts in leaves) which are formed as intermediates like 3-oxo-2(2)-pentenyl-cyclopentane-1-octanoic co-A ligase (3 transcripts in flower, 11 transcripts upregulated in leaves), 12-oxo-phytodienoic acid reductase (2 transcripts in flower, 4 transcripts upregulated in leaves) and 3-oxo-2-(cis-2′-pentenyl)-cyclopentane-1-octanoate (14 transcripts in flower, 60 transcripts in leaves) and leads to the formation of jasmonic acid and followed by three cycles of β-oxidation and finally to *cis* jasmine [29]. From this data it can be hypothesized that the oxylipin pathway transcripts are predominantly upregulated in leaves as compared to the flower.

CYP450 is one of the oldest protein families, has catalytic oxidation function of carbon-carbon bond, alkyl hydroxylation and hydroxyl oxidation, and plays an important role in plant secondary metabolites synthesis process [26]. The data and analysis identified differentially expressed CYP450 genes which can be further assessed for their specific roles in pyrethrin biosynthesis.

In conclusion, the large number of assembled unigenes in both the tissues (flower: 65968; leaf: 80972 sequences) related to pyrethrin biosynthetic pathway derived from *T. cinerariifolium* provides an ideal approach to novel unigenes discovery for a non-model plant that lacks a reference genome. Additionally, the data will be useful in relating the genes associated with the secondary metabolites production and also to analyze the delineation of the functional transcript.

## Conclusion

*De novo* assembly of the transcriptome of *T. cinerariifolium* provides a powerful resource to study biochemical, physiological and genetic processes as well as identification of the metabolic pathways related to pyrethrin biosynthesis in pyrethrum and other related species *viz;* tagetes etc. This study provides the first tissue specific transcripts (flower and leaf) catalogue, which has been generated using 101bp Illumina assembly in pyrethrum. The dataset, including 23.2 million reads for flower and 28.5 million reads for leaf was generated in our study, which can be associated to understand the regulatory cascades and the potential candidate genes, to trace the developmental processes and to study the expression profiles of the transcripts. In this data it has been found that MEP pathway was involved in the biosynthesis of acid moiety of pyrethrin and this pathway predominates in flower as compared to leaf but enzymes related to oxylipin biosynthesis were found predominately in leaf tissue, which suggests that major steps of pyrethrin biosynthesis take place in the flower and this supports the previous studies. Besides pyrethrin biosynthesis, the “omics” (transcriptome and proteome) studies may be applied to derive the beneficial and efficient selection of genotypes owing the desired traits in future.

## Methods

### Plant material

Field grown plants of *Tanacetum cinerariifolium*, from experimental plot of CSIR-CIMAP (Lucknow; Voucher specimen number 12938) field was used for transcriptome analysis. The mature leaves and flowers at full blooming stage were harvested from 2 months old plants and stored in -80° C until used. These samples were further used for RNA extraction.

### RNA isolation and cDNA library construction

Total RNA was extracted from pyrethrum leaves (PYT_L) and flower (PYT_F), using RNeasy Plant Mini kit (Qiagen), according to the manufacturer protocol. The total RNA content was quantified using a Nanodrop spectrophotometer (Nanodrop®, ND-1000, Nanodrop Technologies, and Wilmington, DE, USA). Equal quantity of RNA from samples (PYT_L and PYT_F) was mixed separately for further analysis. To validate the quality of isolated RNA samples were analyzed using Bioanalyzer 2100 (Agilent’s Technology Palo Alto, CA, USA); with [RNA Integrity Number (RIN) values 7.5 and 8] for both PYTF and PYTL respectively. The mRNA library for Illumina sequencing was constructed from 2.4 ug and 3.5 ug of total RNA using Illumina Truseq stranded mRNA sample prep kit (protocol v3) according to manufacturer’s instructions. The average length and quality of cDNA in the library were determined using Agilent’s 2100 Bioanalyzer (Agilent’s Technology). The sequencing and assembly was done by commercial sequencing service provider (NexGenBio, New Delhi, India).

### *De novo* assembly and clustering

Illumina read processing of tissues [flower (PYT_F) and Leaf (PYT_L)] were carried out using 2X101 bp Paired End sequencing on a single lane of the Illumina HIseq 2000 according to manufacturer’s protocol (Genomics Core, UZ Leuven, Belgium). QC and raw data processing were done by FASTQC. After sequencing, the samples PYT_L and PYT_F were demultiplexed and the indexed adapter sequences were trimmed using the CASAVA v1.8.2 software (Illumina, Inc.). Raw reads obtained after RNA-Seq was filtered to remove the sequencing adaptor and low quality reads, using a customer Perl script (CONDETRI: http://code.google.com/p/condetri) with parameters (-hq = 20 -lq = 10 -frac = 0.8 lfrac = 0.1 -minlen = 50 -mh = 5 -ml = 5 -sc = 64) by removing the primer and adapter sequences. Then, the high-quality clean reads were assembled using short read assembler i.e. Trinity rnaseq_r20140413^26^. Percentage of bases, that carry a phred equivalent quality score of 20 and above were assembled into contigs (A score of 20 is equivalent to an error rate of 10^−2^ or an accuracy of 99%). The Data generated and transcripts obtained were deposited at NCBI/Gene Bank as the SRA accession SRP059462.

### Sequence annotation and functional characterization

To annotate the putative function involved in the secondary metabolic pathway, various databases were used to assign the sequence and functional similarity between the candidate genes. The assembled sequence file obtained by Trinity was subjected to PerlCyc database of PMN (Plant Metabolic Network). The Plant Metabolic Network (PMN) provides a broad network of plant metabolic pathway databases that contain curated information from the literature and computational analyses about the genes, enzymes, compounds, reactions, and pathways involved in primary and secondary metabolism in plants. It was observed that only 10-20% of the contigs were annotated i.e. only a fraction of secondary metabolites were obtained. In order to annotate the remaining 80% contigs, they were manually annotated using BLASTX program with NR plant database as reference. The nr database is a ‘non-redundant’ database (i.e. with duplicated sequences removed). NR contains non-redundant sequences from GenBank translations (i.e. GenPept) together with sequences from other databanks (Refseq, PDB, SwissProt, PIR and PRF). The contigs of flower and leaf libraries were annotated against NCBI (nr) (Nr, http://www.ncbi.nlm.nih.gov/) non-reductant GenBank database with BLASTX program. Functional annotation was done by gene ontology softwares (GO; http://www.geneontology.org) and all unigenes were annotated against various protein databases such as UniRef, PFAM, *Simple Modular Architecture Research Tool* (SMART), Kyoto Encyclopedia of Genes and Genomes database (KEGG; http://www.genome.jp/kegg/), NCBI nr GenBank database using Blast X algorithm with e-value threshold of 1e-5. The domains and family search were conducted against Clusters of Orthologous Groups (COG) databases with BLAST programme (E-value < 1e-5). In case, the resultant alignments are in conflict from different databases, the order of preference for best annotation was NCBI nr database results > UniRef > SMART > KEGG. The Blast2GO programme was used for further annotation of unigenes, according to biological function, molecular function and cellular component ontologies (http://www.geneontology.org/). KEGG database was used to annotate the secondary metabolite pathway. To assign putative functions to each unigenes of both leaf and flower tissues, gene ontology analysis (GO) analysis was carried out for the novel discovered transcripts. Go analysis was performed under the categories of biological function, molecular function and cellular components. Then, Web Gene Ontology Annotation *Plot* [WEGO (http://wego.genomics.org.cn/cgi-bin/wego/index.pl)] was used to draw both the GO classification and GO tree. The annotated sequence may belong to one or more GO term, classified to the different functions (molecular function, cellular function and biological function).

### Digital gene expression profiling

The FPKM values for differentially expressed transcripts were determined using a formula FPKM = [Number of reads mapped × 10^9^]/[length of the transcript × total number of reads obtained]. In order to compare the expression levels of the potentially candidate transcripts, it is necessary to normalize the read counts. The number of fragments per kilobase of exon per million fragments mapped reads has been proposed to be powerful metric to normalize the sequence yield and variation in the transcripts length. All the processed reads were mapped to the *Arabidopsis* genome as a reference genome and TopHat (version 2.0.12). Cufflinks (version 2.2.1) was used to estimate the mapped transcripts abundance, by merging the transcripts obtained from flower and leaf tissue respectively. To calculate differential expression of the transcripts; Cuffdiff (version 2.2.1) was used. The down regulation and up regulation of the transcripts were based on the Log 2 fold Change values (log2ratio > 1;up regulated and log2ratio < -1) The differential expression of the up and down regulated transcripts was used in the analysis of pyrethrin metabolite pathway. The genes number of each pathway were calculated by mapping all differentially expressed gene with GO (http://www.geneontology.org/) and KEGG databases (http://www.genome.jp/kegg/).

### Proteome analysis

For proteome analysis; the translated contigs of all samples were used to perform the comparison between different plant species. The proteomes of both the tissues were compared with already published/annotated proteome levels of different model plants like *Oryza sativa*, *Arabidopsis thaliana, Solanum tuberosum*, *Vitis vinifera* and *Sorghum bicolor*.

### HPLC analysis

HPLC analysis of both flower and leaf samples was done to check the presence of pyrethrins respectively. Samples (PYT_L and PYT_F) were harvested at same stage as that for transcriptome analysis and shade dried. The pyrethrins were extracted and analyzed using HPLC as per procedure described by Nagar et. al. [30].

## Additional files

Additional file 1: GO and Pfam ID for PYT_L and PYT_F samples. (XLS 19756 kb)
Additional file 2: List of unigenes of both PYT_L and PYT_F samples with their GO Classification for Biological Process. (XLS 29 kb)
Additional file 3: List of unigenes of PYT_L and PYT_F samples with their GO Classification for Cellular components. (XLS 27 kb)
Additional file 4: List of unigenes of PYT_L and PYT_F samples with their GO Classification for Molecular function. (XLS 27 kb)
Additional file 5: List of tissue specific unigenes assigned to KEGG pathway with their KEGG IDs and Annotation for PYT_L and PYT_F samples. (XLS 18406 kb)
